# Supplementary material for: The causal role of circulating inflammatory markers in osteoporosis: a bidirectional Mendelian randomized study
Source: Front Immunol. 2024 Jul 18;15:1412298. doi: 10.3389/fimmu.2024.1412298 (PMC11291241; doi:10.3389/fimmu.2024.1412298)
Supplement: Supplementary file 1 [file DataSheet_1.zip › supplementary material/CIM-OSTEOPOROSIS /GCST90274782.csv_leave_one_out.pdf]

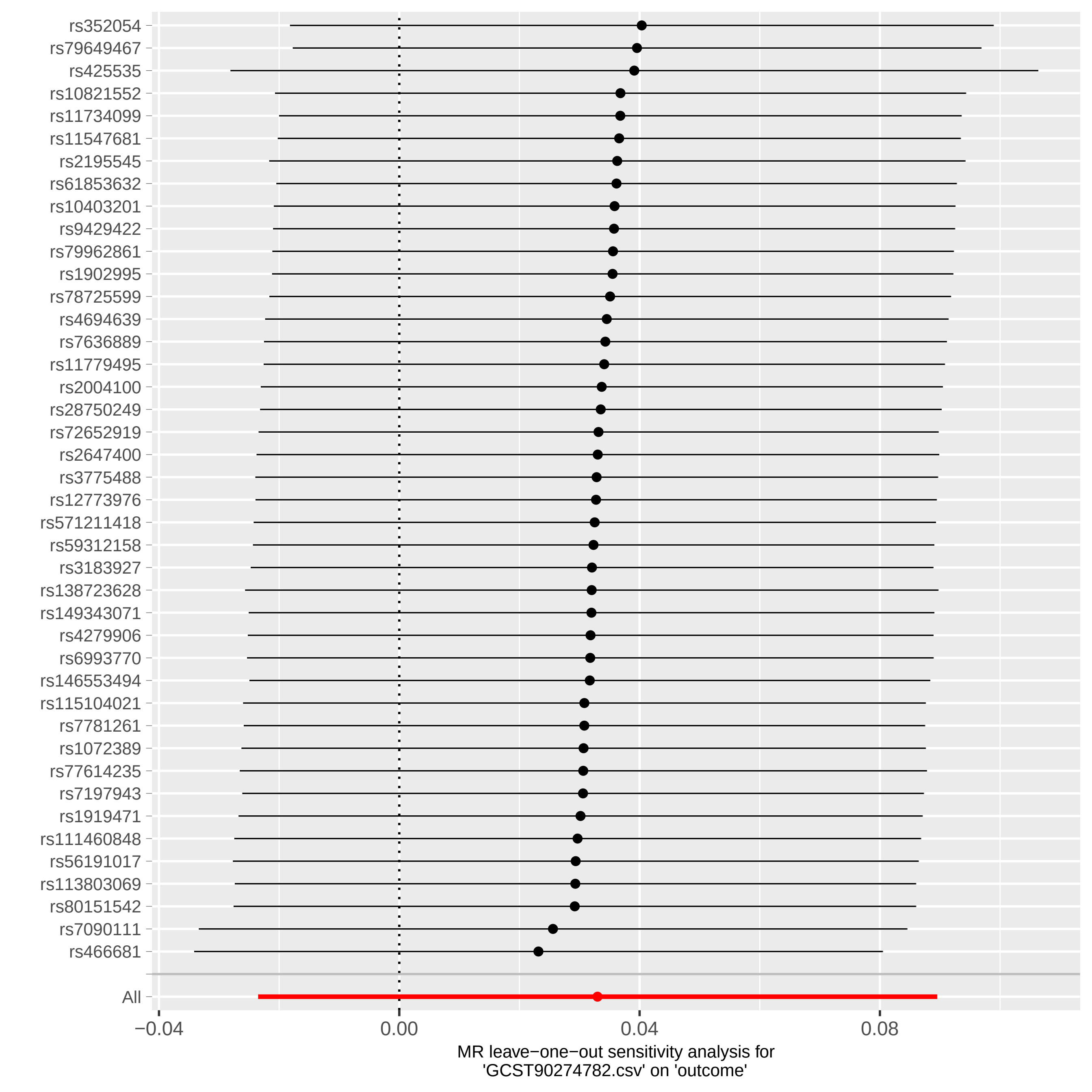

rs352054
rs79649467
rs425535
rs10821552
rs11734099
rs11547681
rs2195545
rs61853632
rs10403201
rs9429422
rs79962861
rs1902995
rs78725599
rs4694639
rs7636889
rs11779495
rs2004100
rs28750249
rs72652919
rs2647400
rs3775488
rs12773976
rs571211418
rs59312158
rs3183927
rs138723628
rs149343071
rs4279906
rs6993770
rs146553494
rs115104021
rs7781261
rs1072389
rs77614235
rs7197943
rs1919471
rs111460848
rs56191017
rs113803069
rs80151542
rs7090111
rs466681
All
−0.04
0.00
0.04
0.08
MR leave−one−out sensitivity analysis for
'GCST90274782.csv' on 'outcome'
